# Characteristics of Community Newspaper Coverage of Tobacco Control and Its Relationship to the Passage of Tobacco Ordinances

**DOI:** 10.1007/s10900-016-0176-8

**Published:** 2016-03-21

**Authors:** Petya Eckler, Shelly Rodgers, Kevin Everett

**Affiliations:** 1 School of Humanities, University of Strathclyde, 141 St James Road, Glasgow, G4 0LT Scotland, UK; 2 School of Journalism, University of Missouri, Columbia, MO USA; 3 School of Medicine, University of Missouri, Columbia, MO USA

**Keywords:** Tobacco control, Newspaper coverage, Public Health Model of Reporting, Public health facts, Content analysis

## Abstract

To answer the call for more systematic surveillance, analysis and evaluation of tobacco news coverage, a 6-year content analysis of newspaper stories from Missouri was conducted to evaluate the presence of public health facts and characteristics of stories framed for or against tobacco control. The method was a content analysis of all Missouri newspapers (N = 381) from September 2006 to November 2011 for a total sample of 4711. Results were connected to the larger, societal context within which newspaper stories reside, i.e., towns that passed or did not pass a smoke-free ordinance during the project intervention. Results showed the majority of news stories were about tobacco control, which were mostly written at the local level, were episodic, and carried a positive slant toward tobacco control. However, there were more negative than positive headlines, and more negative editorials than non-editorials. Tobacco control stories used fewer public health facts than non-tobacco control stories. Towns with existing smoke-free ordinances had more tobacco control stories, and towns without smoke-free ordinances had fewer tobacco control stories and more non-tobacco control stories, suggesting a connection between news media coverage and the passage of smoke-free policies. We conclude that the tobacco industry may have had success in impacting news stories in no-ordinance cities by diverting attention from tobacco control to secondary topics, such as youth smoking, which meant stories had fewer public health facts and fewer positive health benefits in towns that may have needed these details most.

## Introduction

Secondhand smoke causes cancer in humans [1]. Clean indoor air policies, which restrict smoking in certain public spaces (e.g., restaurants), protect people from the harmful effects of secondhand smoke [2]. With tobacco-attributable deaths reaching over 1 billion globally this century, communities around the world are using the mass media to change or initiate smoke-free policies [3–5]. Journalists and editors play a vital role in providing coverage of health issues to the public [6], which may influence whether community members receive adequate and appropriate information about health decisions [7]. Indirect links have been found between tobacco news coverage and tobacco purchase patterns, smoking cessation rates, smoking behaviors, and support for tobacco control policies [8, 9].

While numerous studies have analyzed news coverage of tobacco control, research has yet to connect that to societal factors which may contribute to the type of coverage that communities receive [10]. Towns that have already adopted a smoke-free policy may receive different news coverage than towns that are considering adopting one. Additionally, although research establishes the importance of using public health facts to provide context and perspective on tobacco control issues, scant research has examined the use of public health facts in analyzing tobacco news. Drawing on some public health facts at the expense of others may result in the omission of story details that have relevance for minority and disadvantaged groups [11].

To answer the call for more systematic surveillance, analysis and evaluation of tobacco news coverage [12], a 6-year content analysis of newspaper stories from Missouri was conducted to evaluate the presence of public health facts and characteristics of stories framed for or against tobacco control. All Missouri newspapers (N = 381) were examined from September 2006 to November 2011 for a total sample of 4711 stories. Results were then connected to the larger, societal context within which newspaper stories reside, i.e., towns that had passed or not passed a smoke-free ordinance. Our research was guided by three main questions: (1) How is tobacco control covered by the print media? (2) How are public health facts used in the coverage of tobacco control? (3) Do cities with/without smoke-free ordinances differ in their coverage of tobacco control?

### Framework and Definitions

The Public Health Model of Reporting (PHMR), which promotes a public health perspective on reporting health issues, serves as a useful guide. Our goal is to descriptively assess types of public health information and story characteristics of tobacco news coverage based on this model. PHMR was originally created as a way to spur change in reporting related to crime and violence [13]. However, PHMR has proven useful for other health contexts involving conflict or debate, such as tobacco control [14]. The goal of PHMR is to describe the types of public health facts that would enable readers to gain a broader perspective on issues that may threaten public health [15]. PHMR advocates that part of the solution to public health problems is the provision of critically important information about the causes, costs and consequences of health issues that may prompt individuals to make proactive health choices and, thus, prevent many health problems [16].

### Literature Review

Community newspapers provide information on tobacco issues, such as prevention programs and adoption of smoke-free policies [17–19], and offer a platform for local community members to voice their opinions on the health and economic benefits of smoke-free ordinances [20, 21]. Journalists take a stance on tobacco control ordinances [22], which has been shown to influence their outcome. Of 15 Wisconsin cities with smoke-free ordinance campaigns, all communities that passed ordinances had more news coverage and stronger editorial support from the local newspaper than communities with unsuccessful campaigns [23]. Media coverage of public health issues is a primary way to encourage health-related policy change [24]. Media advocacy, i.e., tactically using mass media to support the public’s ability to organize and advance public health policies [8], has been used to achieve policy success related to tobacco and alcohol control [25].

Frames, which journalists use to communicate complex issues by highlighting some aspects of a particular problem [26], make complex health issues and topics such as tobacco control more salient to readers and can shape the direction of the debate [27]. A national content analysis concluded that stories about tobacco control were framed primarily in terms of political debate rather than public health impact [18]. Tobacco control frames—whether positive/negative or for/against smoke-free ordinances—may influence public opinions or attitudes developed during the formation and discussion of public policy [28]. Smoke-free policies/secondhand smoke and economic issues received the most coverage [29] and provoked the most controversy [29, 30], primarily expressed through editorials and letters to the editor [31, 32].

A number of studies have examined how local and national media cover tobacco control issues, with some consistent emerging trends. Overall, coverage was predominantly positive or neutral/mixed for tobacco control [22, 30, 33–35], which applied to both news stories and editorials, although some studies have found higher negativity in editorials and letters to the editor [22, 35, 36]. Stories with a positive tobacco control slant had information about enforcement, emphasized the lack of negative economic consequences or the health and economic benefits of policies or worker protection [30, 34]. Negatively slanted articles discussed economic losses and hardships, government intrusion and overreach, inability to enforce policies, and smokers’ rights and individual rights [30, 34]. Other popular frames include non-smokers’ rights, smokers’ rights, business rights and health issues [27, 35].

## Method

### Method and Sample

Missouri’s failure to enact a statewide smoke-free policy provides a unique context in which to examine how journalists framed news coverage, and whether this coverage relates to a town’s presence or absence of such policy, as a way to inform other states attempting to pass tobacco legislation. All Missouri newspapers (*N* = 381) were coded over a 6-year period from September 2006 until November 2011. A professional clipping service was hired and keywords were provided to ensure all relevant newspaper stories and editorials were captured. A total of 4711 stories were collected which represented the universe of news stories/editorials for the specified time period. Since the content analysis is a census, sampling variation is zero.

### Coding Categories

There were eight main coding categories: (1) topic, (2) story type, (3) locale, (4) slant of event, (5) slant of opinion, (6) story tone, (7) headline tone, and (8) public health facts.

#### Topic

Topic was defined as the predominant subject of the news story. There were 6 options: (1) health effects of smoking; (2) smoking restrictions or ordinances; (3) anti-smoking programs/services; (4) cigarette taxes; (5) youth smoking issues; and, (6) other. Topic was coded by examining the headline first. For example, if the headline was about “cigarette taxes,” the “cigarette taxes” topic was selected. In cases where the headline was ambiguous or the coder was unsure which topic to code, the first sentence (and no more than the first paragraph) of the story was read to make the determination. See Table 1 for operational definitions.

**Table 1:** Summary of topic definitions

Topic	Description
Health effects of smoking	Smoking as damaging to health; smoking as cause of death (includes cancer, emphysema, etc.); scientific findings that tobacco use has positive/negative effects
Smoking restrictions or ordinances	Effects and enforcement of indoor or outdoor smoking ordinances; smoking bans; smoking policies, including politics or decision groups who meet to make decisions about smoking restrictions (e.g., City Council meetings, etc.)
Anti-smoking programs/services	Cessation and prevention programs; where money for anti-tobacco efforts comes from; anti-smoking ad campaigns; anti-smoking groups/events
Cigarette taxes	Raising the taxes on cigarettes, effect of cigarette price hikes
Youth smoking issues	Youth smoking as distinct from smoking by the general population; smoking bans that focus specifically on youth tobacco activities, school suspensions, etc.; criminalizing the selling of tobacco to youth, restricting self-service, etc.
Other	These included tobacco farming, lawsuits, product issues, tobacco advertising, tobacco consumption by the public, etc.

#### Story Type

Story type referred to the primary format of the news coverage. There were three story types: (1) *event/issue* was defined as a story that is happening currently or recently happened, i.e., “hard news”; (2) *feature* was defined as a special story or article that is not hard news and was distinguished by the style of writing, which was more upbeat; and, (3) *editorial* was defined as an opinionated piece, such as a letter to the editor or an op-ed.

#### Locale

Locale was defined as the geographic place in which the story’s event, issue, feature or editorial took place. There were four categories: (1) local (occurred in the town in which the newspaper is located), (2) state (occurred in the state in which the newspaper resides), (3) national (occurred anywhere else in the U.S. except for the state in which the newspaper resides), and (4) international (occurred outside of the U.S.). Upon inspection, too few cases were international (n = 40) so they were deleted from further analysis.

#### Slant of Event

Event slant referred to the underlying assumptions, beliefs and ideologies that can serve as heuristics for how a particular news story could be understood. Here, the focus was on how the news story presented information for and against tobacco control for the purposes of framing tobacco news. There were four options: (1) positive for tobacco control; (2) negative for tobacco control; (3) mixed impact on tobacco control (4) N/A. Positive for tobacco control was defined as stories that supported additional education, regulation or restriction on the tobacco industry. Any event where the position of the tobacco industry was upheld (e.g., not to pass clean indoor air ordinances) was coded as “negative for tobacco control”. Mixed impact on tobacco control was defined in terms of events or issues brought up by the writer that have some good news and some bad news in relation to tobacco control.

#### Slant of Opinion

Opinion pieces were coded for slant using the same definitions and criteria identified above for *Slant of event*. The categories were: (1) positive for tobacco control, (2) negative for tobacco control, and (3) mixed impact on tobacco control.

#### Story Tone

Story tone was defined as the primary “attitude” or valence of the story. There were three choices: (1) positive, (2) negative, and (3) neutral. A positive tone consisted of viewpoints on the positive consequences of tobacco control ordinances. A negative tone consisted of viewpoints on the negative consequences of tobacco control ordinances. A neutral tone was defined as neither predominantly positive nor negative, i.e., the tone was factual with no apparent viewpoint for or against tobacco control policies. The procedure used by Cohen et al. [37] was applied to determine story tone.

#### Headline Tone

Headline tone was defined as the primary “attitude” or valence of the headline. Options included: (1) positive, (2) negative, and (3) neutral. The same definitions and procedures described in *Story tone* were used to code headline tone, except that individual words in headlines served as the unit of analysis.

#### Public Health Facts

Three types of public health (PH) facts about tobacco and secondhand smoke were coded: context, costs, and consequences [13]. *Context* was defined as PH facts with comparisons or contrasts, e.g., comparing smoking rates across communities, comparing cigarette taxes across states, or comparing two or more groups. Comparisons were both statistical and narrative. *Costs* were economic expenses due to PH problems at familial, community, city, state, or national levels. These included societal expenses from tobacco-related illnesses, individual costs to smokers, and state costs of secondhand smoke. *Consequences* were the outcome or impact of tobacco and secondhand smoke issues. Examples included health effects of quitting smoking, effects of secondhand smoke on employees, or consequences of smoking ordinances. PH facts were coded as ratio-level variables, so a newspaper story could have many PH facts of all types, thus providing a more complex picture.

### Unit of Analysis and Reliabilities

The unit of analysis was the news story. Public health facts were determined by examining individual sentences within news stories. Headline tone was determined by examining individual words in headlines. Tone and slant were determined by examining individual sentences in stories/opinion pieces. Five graduate students were the coders. The codebook was pre-tested by two initial coders. Inter-coder reliabilities were taken at the beginning, middle and end of the project to ensure coding was reliable and had not shifted during the coding process. An overall Scott’s pi [38] index of .82 was reached (range .60–.89) for nominal variables. Observed agreement was calculated at .89 (range .60–1.0) for the ratio-level PH facts.

### Additional Data

A new column was added to our content analysis data set, indicating whether cities had passed (1) or not passed (2) a clean indoor air ordinance during the 6-year content analysis period.

### Statistical Analyses

Chi square tests of independence were conducted to examine the research questions. Statistical significance was tested at the 95 % confidence level. Statistical analyses were performed using SPSS version 23.

## Results

### RQ1: How was Tobacco Control Covered by the Print Media?

A total of 4671 stories on tobacco were analyzed (see Table 2), 62.7 % of those (n = 2929) were about tobacco control (TC): smoke-free ordinances or cigarette taxes/prices. The remaining 37.3 % of stories were about the health effects of smoking, anti-smoking programs/services, youth smoking issues, and others. TC was covered primarily on a local level, as 48.9 % of stories had a local angle and 41.5 % had a state angle. This differed from non-TC stories, which were primarily written with a state-wide focus (49.3 %) and had fewer local stories (30.4 %). See Table 3.

**Table 2:** Overall coverage of tobacco in Missouri newspapers

	N	%
Story type		
Event/issue	3174	68
Editorial	1432	30.7
Feature	65	1.4
Story locale		
State	2075	44.4
Local	1962	42
National	634	13.6
Story topic		
Smoking ordinances	2548	54.5
Anti-smoking programs	650	13.9
Other	464	9.9
Cig. taxes/prices	381	8.2
Youth issues	347	7.4
Health effects	281	6
Context facts		
0 facts	3369	72.1
1 fact	650	13.9
2 facts	287	6.1
≥3 facts	365	7.8
Costs facts		
0 facts	3913	83.8
1 fact	423	9.1
2 facts	189	4
≥3 facts	146	3.1
Consequences facts		
0 facts	3628	77.7
1 fact	503	10.8
2 facts	243	5.2
≥3 facts	297	6.4

N = 4671

**Table 3:** Coverage of tobacco control versus non-tobacco control topics in Missouri newspapers

	Tobacco control stories		Non-tobacco control stories	
	N	%	N	%
Story type				
Event/issue	1687	57.6	1487	85.4
Editorial	1222	41.7	210	12.1
Feature	20	.7	45	2.6
Story locale				
State	1216	41.5	859	49.3
Local	1433	48.9	529	30.4
National	280	9.6	354	20.3
Story topic				
Smoking ordinances	2548	87	650	37.3
Cig. taxes/prices	381	13	347	19.9
Anti-smoking programs			281	16.1
Youth issues			464	26.6
Health effects				
Other				
Context facts				
0 facts	2140	73.1	1229	70.6
1 fact	393	13.4	257	14.8
2 facts	182	6.2	105	6
≥3 facts	214	7.3	151	8.7
Costs facts				
0 facts	2604	88.9	1309	75.1
1 fact	173	5.9	250	14.4
2 facts	86	2.9	103	5.9
≥3 facts	66	2.3	80	4.6
Consequences facts				
0 facts	2426	82.8	1202	69
1 fact	261	8.9	242	13.9
2 facts	130	4.4	113	6.5
≥3 facts	112	3.8	185	10.6
	n = 2929		n = 1742	

N = 4671

TC stories were primarily events/issues (57.6 %) followed by editorials/letters to the editor (41.7 %) and very few features (.7 %). Many more editorials were written on TC topics (41.7 %) versus non-tobacco control ones (12.1 %).

Of the event/issue/feature stories about TC, 52.9 % had a positive slant for TC and 37.2 % had mixed slant. Only 9.9 % upheld the position of the tobacco industry. Of editorials on TC issues, 53.6 % had a positive slant for TC, 11.3 % had a mixed impact on TC, and 35.2 % upheld the position of the tobacco industry.

In terms of tone of TC coverage, the vast majority of stories had a neutral tone (99.2 %) and a slightly lower number presented neutral headlines (93 %). However, 5.5 % of stories did run with negative headlines, compared to only 1.1 % with positive ones. For editorial content on TC, the tone of the text and headline continued to be predominantly neutral (86.3 % for stories and 84.9 % for headlines). However, a larger presence of negativity was noticed in both copy and headlines (12.2 % for copy, 11.4 % for headlines).

### RQ2: How were Public Health Facts Used in the Coverage of Tobacco Control?

The ratio variable PH facts had high skewness and kurtosis and lacked normal distribution, which necessitated its transformation into a categorical variable. Four categories of facts were created: 0 facts, 1 fact, 2 facts, ≥3 facts for each type of PH fact. Chi square tests were applied to test for relationships with other variables.

TC stories used fewer public health facts compared to non-TC stories. For TC stories, there were .58 context facts on average, compared to .68 for non-TC stories; .22 costs facts on average, compared to .46 for non-TC stories; and .35 consequence facts on average compared to .81 in non-TC stories. Differences were significant for costs (*χ*^*2*^ = 153.75, df = 3, *p* < .001) and consequences (*χ*^*2*^ = 140.21, df = 3, *p* < .001).

When TC coverage was examined on its own, several relationships emerged between the use of public health facts and the stories’ locale, type, and slant. Chi square tests were used to check for differences between expected and observed frequencies of public health facts. Standardized residuals helped examine for differences in each cell of the test. When those residuals were greater than ±1.96, differences in the cell were deemed significant.

A significant relationship existed between locale and number of facts ($\chi_{\text{context}}^{2}$ = 41.15, df = 6, *p* < .001; $\chi_{\text{costs}}^{2}$ = 78.82, df = 6, *p* < .001; and $\chi_{\text{conseq}}^{2}$ = 18.75, df = 6, *p* = .005). Standardized residuals showed that more state stories contained 1 and 2 context facts, while fewer local stories had 2 facts than expected. On costs, local stories had fewer cost facts than expected, while state stories had more than expected. On consequences, only national stories had more 2 and 3 facts than expected.

A significant relationship existed between story type and number of facts for context and costs ($\chi_{\text{context}}^{2}$ = 26.69, df = 3, *p* < .001; $\chi_{\text{costs}}^{2}$ = 21.88, df = 3, *p* < .001). Standardized residuals showed that there were more news/features with 2 context facts than expected and fewer editorials with 2, 3 or more facts than expected. There were more news/features with 1 cost fact than expected and fewer editorials with 1 cost fact than expected.

There was a significant relationship between the slant of events towards TC and the number of PH facts used ($\chi_{\text{context}}^{2}$ = 48.71, df = 9, *p* < .001; $\chi_{\text{costs}}^{2}$ = 36.35, df = 9, *p* < .001; and $\chi_{\text{conseq}}^{2}$ = 18.04, df = 9, *p* = .035). Standardized residuals showed that events that were positive for TC were covered with more context facts than expected, while events that were negative for TC had fewer stories with 1 fact than expected. Events with mixed slant on TC were covered with more stories with 1 and 2 cost facts than expected and more stories with 3 or more consequence facts than expected.

There was also a significant relationship between the slant of opinion towards TC and the number of PH facts used ($\chi_{\text{context}}^{2}$ = 107.12, df = 9, *p* < .001; $\chi_{\text{costs}}^{2}$ = 35.01, df = 9, *p* < .001; and $\chi_{\text{conseq}}^{2}$ = 51.99, df = 9, *p* ≤ .001). Editorials with a positive slant for TC had more stories than expected with 1 context fact and those that withheld the position of the tobacco industry had fewer stories than expected with any number of context facts. For costs facts, fewer editorials than expected with both positive and negative slant for TC contained 1 fact. For consequences, more opinion pieces with positive slant than expected contained 1 and 2 facts, while fewer opinion pieces with negative slant contained 1 and 3 or more facts.

### RQ3: Did Cities With/Without Smoke-Free Ordinances Differ in Their Coverage of Tobacco Control?

Of the overall number of stories on tobacco in Missouri (N = 4671), 51.2 % was published in towns/cities with smoke-free ordinances. There was a significant difference in the number of TC stories published in towns with a smoke-free ordinance and without one (χ^2^ = 412.9, df = 1, *p* < .001). Towns with an ordinance had more stories on TC in their newspapers and fewer stories on other tobacco topics than expected. Towns without a smoking ordinance had fewer stories on TC but more stories on other tobacco topics.

There was a significant difference in the types of stories published in towns with smoke-free ordinance versus towns without (χ^2^ = 159.59, df = 1, *p* < .001). In towns with ordinances, more editorials and fewer news stories were published than expected. On the contrary, in towns without ordinances, there were more news stories and fewer editorials. The tobacco-related topics that dominated the newspaper coverage also differed (χ^2^ = 506.55, df = 5, *p* < .001). In towns with ordinances, stories about smoke-free ordinances and cigarette taxes (i.e., stories on TC) were more common than expected, while the remaining topics appeared less often than expected. In towns without ordinances, the opposite was observed: more stories than expected on smoking effects, anti-smoking programs and youth issues and fewer stories than expected on tobacco control topics.

Last, the relationship between presence of ordinances and PH facts was examined and found significant for costs (χ^2^ = 36.69, df = 3, *p* < .001) and consequences (χ^2^ = 29.14, df = 3, *p* < .001). In towns with ordinances, fewer stories than expected contained 1 cost fact and 2, 3 or more consequence facts. In towns without ordinances, more stories than expected contained 1 cost fact and more stories than expected contained 2, 3 or more consequence facts.

## Discussion

Journalists and editors play a vital role in providing coverage of health topics to the public [6], which may influence whether communities receive appropriate information to influence health decisions [7]. Our results revealed that tobacco control was the most prevalent of all examined topics, taking more than half of the coverage. Positive slants that supported tobacco control were revealed in slightly over half of all stories, and more than one-third of stories had mixed slants (some for and some against). A high percentage of neutral editorials that did not have extreme positive or negative language was identified, suggesting an attempt by journalists and editors to provide balanced coverage. Results revealed that news stories and editorials on tobacco control contained very few public health facts overall; and, more than half of the coverage focused on events, which tend to be episodic in nature. This suggests that Missouri readers may have received bits and pieces of the issue of tobacco control rather than a seamless and on-going elaboration. Close to half of stories on tobacco control were local, which reflects the fact that in Missouri the battle for smoking restrictions occurred in individual communities and not on the state level. Local stories that contain information tailored to a particular group would benefit readers by providing a more relevant perspective on tobacco-related issues affecting their communities.

In terms of story tone, our results showed that tobacco control stories had more negative than positive headlines, and tobacco control editorials were more negative than non-editorial content. This may suggest that editors were cognizant of the predominantly positive or mixed slant of non-editorial content and attempted to balance coverage by providing more negatively slanted editorials. Alternatively, it may be that there were more editorials written by individuals who, by and large, did not support smoke-free policies, which subsequently gave them a bigger “voice” on the editorial pages of the newspapers. Further, many more editorials were written on tobacco control topics than non-tobacco control ones, showing once again that smoking ordinances and cigarette taxes are very popular, and often controversial, topics which ignite public opinion.

Guided by the PHMR, results showed that tobacco control stories used fewer public health facts than non-tobacco control stories, and public health facts tended to be provided at a state (rather than local) level and consisted of context facts used in event (episodic) stories. Stories that were predominantly against tobacco control used fewer public health facts, suggesting that tobacco advocates (and journalists who covered the stories) did not rely on public heath facts to make their case. Overall, towns with smoking ordinances received coverage with fewer public health facts.

These findings paint a more complex picture than previously known by showing that there are layers of the narrative provided by newspapers in communities where everyday citizens have a voice through editorial coverage. The most dominant topic was tobacco control or restrictions. However, there were smaller “voices” in the background that discussed secondary issues, such as youth smoking, anti-smoking programs, and towns that had not adopted clean indoor air ordinances sometimes got side-tracked discussing such subsidiary topics. This suggests that tobacco advocates (who were also writing editorials during this time) may have succeeded in diverting attention from the primary issue of adopting clean indoor air policies to secondary issues. Additionally, our results suggest that editorials with negative tones were published in cities with high smoking rates and no ordinances, presumably because they wanted to maintain the status quo and not pass a clean indoor air ordinance.

### Theoretical Implications

Much of public knowledge about health problems associated with secondhand smoke comes from the news media. The PHMR provides a theoretical context in which to understand frames and public health facts associated with tobacco control coverage. Our results showed that coverage may not be living up to the standards set forth by the PHMR. Of the three types of public health facts examined, context facts were most prevalent. Additionally, while we might expect that tobacco control stories would highlight the positive health benefits of enacting smoke-free policies, our results revealed the opposite—there were fewer public health facts present in tobacco control as compared to non-tobacco control stories. Towns that already had passed a smoke-free ordinance had fewer public health facts overall. Reporters were also more likely to use episodic coverage and avoid public health facts that would likely provide context and depth to help readers understand the health problems associated with secondhand smoke.

### Practical Implications

These findings are useful for media advocates who seek to inform communities through exposure to targeted messages, and mobilize them to influence policy makers and voters to reform policy. The findings also offer practical suggestions for health and strategic communicators and those involved in public policy, as news stories and editorials can be used to bring about health related and public policy changes to communities. Specifically, health communicators should pay more attention to editorials, where much of the debate about policy changes occurred. Editorials contained higher levels of negativity than news stories and were used actively by proponents of the tobacco industry to oppose restrictive measures. Therefore, health advocates need specific strategies addressing editorial content in local newspapers when planning tobacco control campaigns. Health advocates should also provide relevant local information to journalists, as findings showed that coverage of tobacco control was predominantly local. Further, a higher use of public health facts about the consequences and costs of secondhand smoke could provide more persuasive power to news stories and opinions. The reasons for introducing smoke-free ordinances are the harmful effects of secondhand smoke and the associated healthcare and societal costs. Yet, these reasons are rarely mentioned in stories about ordinances, and using more public health facts on costs and consequences could address that. Finally, towns without smoke-free ordinances which are considering policy changes would benefit from an early visibility of the issue in the local print media, as people in these towns rely on local newspapers for their health news [39]. That would set the agenda and drive the issue forward, as well as could help health advocates outline the key benefits of such policies from the start. Local newspapers have been shown to be important for informing small communities (and city leaders) about the pulse and voice of where residents stand on a given issue.

### Limitations and Future Directions for Research

One of the strengths of this study was the ability to relate content analysis results back to the environmental/societal context in which they appear, i.e., the cities in which the newspapers reside. However, causality cannot be established using content analysis data. Additionally, a content analysis cannot tell us about the intentional or unintentional (i.e., gatekeeping) selections of journalists, editors or publishers of the newspapers. Although the total number of public health facts was counted in each story, we limited the number of public health facts to three main types, which may not have captured other types that may be present. Last, while we assume that public health facts have an impact on public understanding of health issues and on individuals’ health behaviour, our study cannot determine whether this is the case. Future studies can remedy these limitations.

## Conclusion

This study found that the public health perspective was not that important to the discussions and reporting of tobacco control in Missouri for the time period examined. While the majority of newspaper coverage was neutral or positive toward tobacco control, the tobacco industry may have had success in impacting news stories in no-ordinance cities by diverting attention from tobacco control to secondary topics such as youth smoking, which meant fewer chances to highlight the health benefits of smoke-free policies in towns that may have needed it most.
